# Crystal structure of (4*Z*)-4-[(di­methyl­amino)­methyl­idene]-3,5-dioxo-2-phenyl­pyrazolidine-1-carbaldehyde

**DOI:** 10.1107/S2056989015010038

**Published:** 2015-06-03

**Authors:** Joel T. Mague, Shaaban K. Mohamed, Mehmet Akkurt, Eman A. Ahmed, Ahmed Khodairy

**Affiliations:** a Department of Chemistry, Tulane University, New Orleans, LA 70118, USA; b Chemistry and Environmental Division, Manchester Metropolitan University, Manchester M1 5GD, England; c Chemistry Department, Faculty of Science, Minia University, 61519 El-Minia, Egypt; d Department of Physics, Faculty of Sciences, Erciyes University, 38039 Kayseri, Turkey; e Chemistry Department, Faculty of Science, Sohag University, 82524 Sohag, Egypt

**Keywords:** crystal structure, pyrazolo­nes, short intra­molecular C—H⋯O contact, C—H⋯O hydrogen bonds, C—H⋯π inter­actions

## Abstract

In the title compound, C_13_H_13_N_3_O_3_, the pyrazolidine ring adopts a shallow envelope conformation, with the carbonyl C atom closest to the benzene ring as the flap [deviation of 0.126 (1) Å from the plane through the remaining atoms (r.m.s. deviation = 0.011 Å)]. The dihedral angle between the pyrazolidine ring (all atoms) and the benzene ring is 51.09 (4)°. An extremely short (2.08 Å) intra­molecular C—H⋯O contact is seen. In the crystal, mol­ecules are linked by C—H⋯O bonds, generating [010] chains. Extremely weak C—H⋯π inter­actions are also observed.

## Related literature   

For biological studies of azole compounds, see: Patel *et al.* (2012 ▸); Vijesh *et al.* (2011 ▸). For various medicinal and industrial applications of pyrrazole-containing compounds, see: Jin *et al.* (2011 ▸); Zhang *et al.* (2010 ▸); El-Sabbagh *et al.* (2009 ▸); Dekhane *et al.* (2011 ▸); Rostom *et al.* (2003 ▸); Zhou *et al.* (2010 ▸); Finkelstein & Strock (1997 ▸).
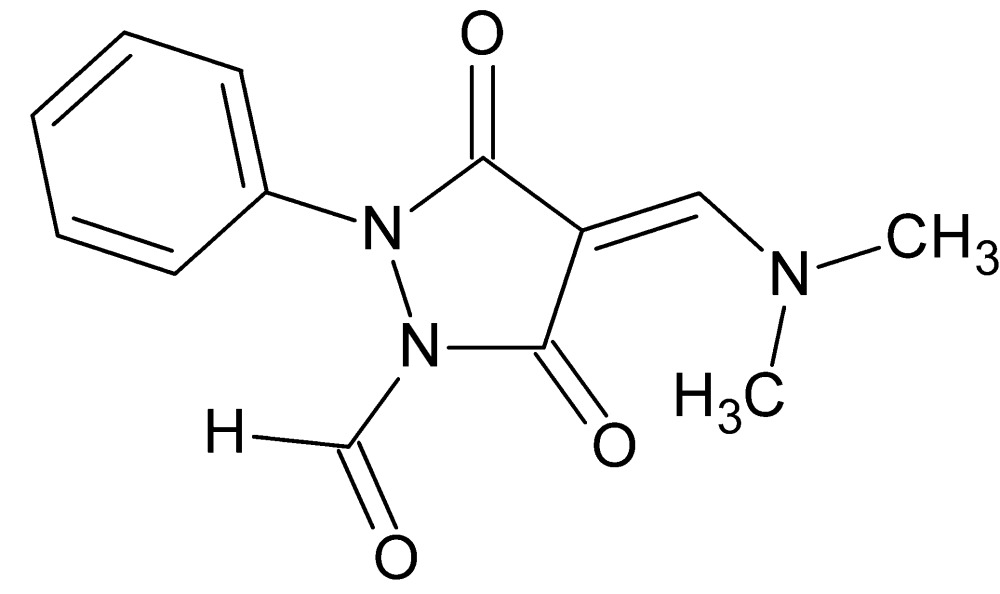


## Experimental   

### Crystal data  

C_13_H_13_N_3_O_3_
*M* *_r_* = 259.26
Monoclinic, 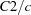

*a* = 26.4235 (9) Å
*b* = 6.1033 (2) Å
*c* = 16.8611 (6) Å
β = 113.272 (1)°
*V* = 2497.96 (15) Å^3^
*Z* = 8
Cu *K*α radiation
μ = 0.84 mm^−1^
*T* = 150 K
0.24 × 0.15 × 0.05 mm

### Data collection  

Bruker D8 VENTURE PHOTON 100 CMOS diffractometer
Absorption correction: multi-scan (*TWINABS*; Sheldrick, 2009 ▸) *T*_min_ = 0.82, *T*_max_ = 0.96
26486 measured reflections
4565 independent reflections
3979 reflections with *I* > 2σ(*I*)
*R*_int_ = 0.021

### Refinement  

*R*[*F* ^2^ > 2σ(*F* ^2^)] = 0.037
*wR*(*F* ^2^) = 0.104
*S* = 1.04
4565 reflections
175 parameters
H-atom parameters constrained
Δρ_max_ = 0.26 e Å^−3^
Δρ_min_ = −0.20 e Å^−3^

### 

Data collection: *APEX2* (Bruker, 2014 ▸); cell refinement: *SAINT* (Bruker, 2014 ▸); data reduction: *SAINT*; program(s) used to solve structure: *SHELXT* (Sheldrick, 2015*a* ▸); program(s) used to refine structure: *SHELXL2014* (Sheldrick, 2015*b* ▸); molecular graphics: *DIAMOND* (Brandenburg & Putz, 2012 ▸); software used to prepare material for publication: *SHELXTL* (Sheldrick, 2008 ▸).

## Supplementary Material

Crystal structure: contains datablock(s) global, I. DOI: 10.1107/S2056989015010038/hb7425sup1.cif

Structure factors: contains datablock(s) I. DOI: 10.1107/S2056989015010038/hb7425Isup2.hkl

Supporting information file. DOI: 10.1107/S2056989015010038/hb7425Isup3.cml

**Fig. 1.**
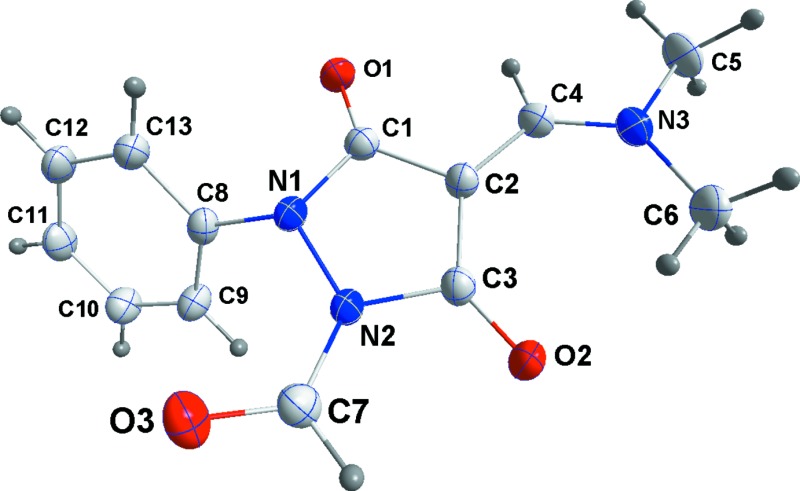
The title molecule with 50% probability ellipsoids.

**Fig. 2.**
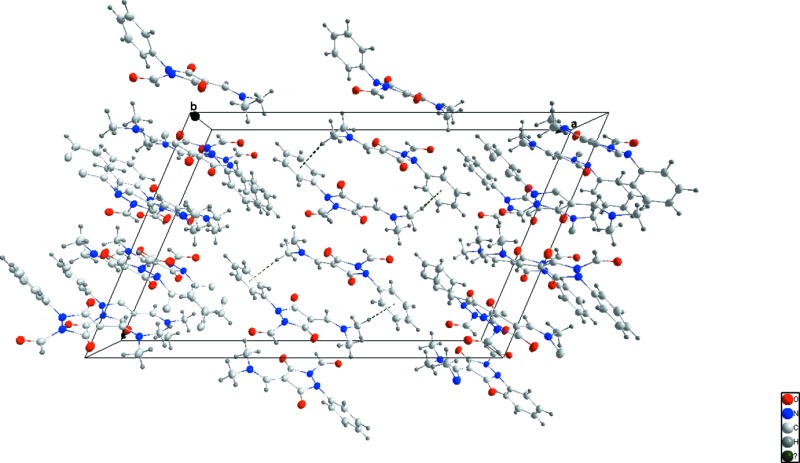
Packing viewed down the b axis. C—H···π interactions are shown by dotted lines.

. DOI: 10.1107/S2056989015010038/hb7425fig1.tif
The title mol­ecule with 50% probability ellipsoids.

b . DOI: 10.1107/S2056989015010038/hb7425fig2.tif
Packing viewed down the *b* axis. C—H⋯π inter­actions are shown by dotted lines.

CCDC reference: 1402532

Additional supporting information:  crystallographic information; 3D view; checkCIF report

## Figures and Tables

**Table 1:** Hydrogen-bond geometry (, ) *Cg*1 is the centroid of the C1/C2/C3/N1/N2 ring.

*D*H*A*	*D*H	H*A*	*D* *A*	*D*H*A*
C6H6*A*O2	0.98	2.08	3.016(2)	160
C5H5*C*O3^i^	0.98	2.52	3.1803(19)	124
C7H7O2^ii^	0.95	2.29	3.0663(16)	139
C5H5*B* *Cg*1^iii^	0.98	2.98	3.8823(18)	153

Symmetry codes: (i) 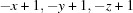; (ii) 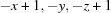; (iii) 
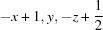
.

## supplementary crystallographic information

### S1. Comment

Azole compounds are extensively studied and widely used as anti-microbial agents (Patel *et al.*, 2012; Vijesh *et al.*, 2011). Recently, urea derivatives of pyrazole have been reported as potent inhibitors of p38 kinase (Jin *et al.*, 2011). Many of other pyrazole scaffold compounds are reported to have broad spectra of biological activities, such as anti-fungal (Zhang *et al.*, 2010), anti-viral (El-Sabbagh *et al.*, 2009), anti-inflammatory (Dekhane *et al.*, 2011), anti-tumor, anti-HCV (Rostom *et al.*, 2003), herbicidal (Zhou *et al.*, 2010) and insecticidal activities (Finkelstein & Strock, 1997). In view of such findings and as a continuation of our study on the synthesis of potential bio-active heterocyclic molecules, we report here the synthesis and crystal structure of the title compound.

In the title compound, the pyrazolidine ring is slightly twisted with an r.m.s. deviation from the mean plane of the 5 atoms forming the ring of 0.036 Å. The dihedral angle between this plane and that of the phenyl ring is 51.09 (4)° (Fig. 1).

### S2. Experimental

To phosphorous oxychloride (0.1 mol, 10 ml), in a conical flask with a magnetic stirrer, dry dimethylformamide (35 ml) was added drop-wise with stirring at 303–308 K for 30 min. Then a solution of 1-phenylpyrazolidine-3,5-dione (0.05 mol, 8.8 g) in dimethylformamide (15 ml), was added drop-wise with continuous stirring while ensuring that the temperature did not exceed 318 K. The reaction mixture was stirred overnight and poured onto crushed ice. The solid product was collected by filtration and recrystallized from ethanol to give colourless crystals in 75% yield (m. p. 453–455 K).

### S3. Refinement

H-atoms were placed in calculated positions (C—H = 0.95 - 0.98 Å) and included as riding contributions with isotropic displacement parameters 1.2 - 1.5 times those of the attached carbon atoms. The model was refined as a 2-component twin.

### Crystal data

**Table:**

C_13_H_13_N_3_O_3_	*F*(000) = 1088
*M**_r_* = 259.26	*D*_x_ = 1.379 Mg m^−^^3^
Monoclinic, *C*2/*c*	Cu *K*α radiation, λ = 1.54178 Å
*a* = 26.4235 (9) Å	Cell parameters from 9978 reflections
*b* = 6.1033 (2) Å	θ = 3.6–72.3°
*c* = 16.8611 (6) Å	µ = 0.84 mm^−^^1^
β = 113.272 (1)°	*T* = 150 K
*V* = 2497.96 (15) Å^3^	Rod, colourless
*Z* = 8	0.24 × 0.15 × 0.05 mm

### Data collection

**Table:**

Bruker D8 VENTURE PHOTON 100 CMOS diffractometer	4565 independent reflections
Radiation source: INCOATEC IµS micro–focus source	3979 reflections with *I* > 2σ(*I*)
Mirror monochromator	*R*_int_ = 0.021
Detector resolution: 10.4167 pixels mm^-1^	θ_max_ = 72.3°, θ_min_ = 3.6°
ω scans	*h* = −32→30
Absorption correction: multi-scan (*TWINABS*; Sheldrick, 2009)	*k* = −7→7
*T*_min_ = 0.82, *T*_max_ = 0.96	*l* = −20→20
26486 measured reflections	

### Refinement

**Table:**

Refinement on *F*^2^	Primary atom site location: structure-invariant direct methods
Least-squares matrix: full	Secondary atom site location: difference Fourier map
*R*[*F*^2^ > 2σ(*F*^2^)] = 0.037	Hydrogen site location: inferred from neighbouring sites
*wR*(*F*^2^) = 0.104	H-atom parameters constrained
*S* = 1.04	*w* = 1/[σ^2^(*F*_o_^2^) + (0.0577*P*)^2^ + 0.7347*P*] where *P* = (*F*_o_^2^ + 2*F*_c_^2^)/3
4565 reflections	(Δ/σ)_max_ < 0.001
175 parameters	Δρ_max_ = 0.26 e Å^−^^3^
0 restraints	Δρ_min_ = −0.20 e Å^−^^3^

### Special details

**Table:**

Experimental. Analysis of 3764 reflections having I/σ(I) > 12 and chosen fromthe full data set with *CELL_NOW* (Sheldrick, 2008) showedthe crystal to belong to the monoclinic system and to be twinnedby a 180° rotation about the *c** axis. The raw data wereprocessed using the multi-component version of *SAINT* undercontrol of the two-component orientation file generated by*CELL_NOW*.
Geometry. All e.s.d.'s (except the e.s.d. in the dihedral angle between two l.s. planes)are estimated using the full covariance matrix. The cell e.s.d.'s are takeninto account individually in the estimation of e.s.d.'s in distances, anglesand torsion angles; correlations between e.s.d.'s in cell parameters are onlyused when they are defined by crystal symmetry. An approximate (isotropic)treatment of cell e.s.d.'s is used for estimating e.s.d.'s involving l.s. planes.
Refinement. Refinement of *F*^2^ against ALL reflections. The weighted *R*-factor *wR* andgoodness of fit *S* are based on *F*^2^, conventional *R*-factors *R* are basedon *F*, with *F* set to zero for negative *F*^2^. The threshold expression of*F*^2^ > σ(*F*^2^) is used only for calculating *R*-factors(gt) *etc*. and isnot relevant to the choice of reflections for refinement. *R*-factors basedon *F*^2^ are statistically about twice as large as those based on *F*, and *R*-factors based on ALL data will be even larger. H-atoms were placed incalculated positions (C—H = 0.95 - 0.98 Å) and included as ridingcontributions with isotropic displacement parameters 1.2 - 1.5 times thoseof the attached carbon atoms. The model was refined as a 2-component twin.

### Fractional atomic coordinates and isotropic or equivalent isotropic displacement parameters (Å^2^)

**Table:**

	*x*	*y*	*z*	*U*_iso_*/*U*_eq_	
O1	0.43175 (4)	0.79022 (16)	0.28697 (7)	0.0296 (2)	
O2	0.52891 (4)	0.15926 (16)	0.42906 (7)	0.0324 (3)	
O3	0.37052 (4)	0.10698 (18)	0.39957 (6)	0.0316 (3)	
N1	0.40831 (4)	0.46179 (18)	0.33205 (7)	0.0236 (3)	
N2	0.43832 (4)	0.26978 (18)	0.36829 (7)	0.0239 (3)	
N3	0.59876 (5)	0.64368 (19)	0.42207 (8)	0.0265 (3)	
C1	0.44610 (5)	0.6170 (2)	0.32599 (8)	0.0233 (3)	
C2	0.50109 (5)	0.5284 (2)	0.37179 (8)	0.0235 (3)	
C3	0.49589 (5)	0.3060 (2)	0.39467 (8)	0.0242 (3)	
C4	0.54556 (5)	0.6654 (2)	0.38045 (9)	0.0249 (3)	
H4	0.5349	0.8001	0.3502	0.030*	
C5	0.63539 (6)	0.8195 (3)	0.41755 (10)	0.0344 (3)	
H5A	0.6133	0.9445	0.3861	0.052*	
H5B	0.6580	0.7661	0.3874	0.052*	
H5C	0.6594	0.8652	0.4761	0.052*	
C6	0.62712 (6)	0.4598 (3)	0.47712 (10)	0.0344 (3)	
H6A	0.6002	0.3471	0.4750	0.052*	
H6B	0.6462	0.5109	0.5367	0.052*	
H6C	0.6540	0.3978	0.4566	0.052*	
C7	0.41757 (5)	0.1104 (2)	0.40511 (8)	0.0255 (3)	
H7	0.4416	−0.0034	0.4367	0.031*	
C8	0.35475 (5)	0.4373 (2)	0.26388 (8)	0.0235 (3)	
C9	0.34078 (5)	0.2502 (2)	0.21315 (9)	0.0275 (3)	
H9	0.3667	0.1349	0.2227	0.033*	
C10	0.28831 (6)	0.2336 (3)	0.14813 (9)	0.0311 (3)	
H10	0.2783	0.1062	0.1129	0.037*	
C11	0.25040 (6)	0.4019 (3)	0.13433 (9)	0.0311 (3)	
H11	0.2146	0.3899	0.0897	0.037*	
C12	0.26490 (6)	0.5880 (2)	0.18593 (10)	0.0308 (3)	
H12	0.2390	0.7031	0.1765	0.037*	
C13	0.31717 (5)	0.6066 (2)	0.25127 (9)	0.0271 (3)	
H13	0.3271	0.7333	0.2868	0.033*	

### Atomic displacement parameters (Å^2^)

**Table:**

	*U*^11^	*U*^22^	*U*^33^	*U*^12^	*U*^13^	*U*^23^
O1	0.0274 (5)	0.0239 (5)	0.0339 (5)	0.0033 (4)	0.0083 (4)	0.0053 (4)
O2	0.0221 (5)	0.0284 (5)	0.0409 (6)	0.0034 (4)	0.0063 (4)	0.0100 (4)
O3	0.0259 (5)	0.0404 (6)	0.0290 (5)	−0.0054 (4)	0.0115 (4)	0.0019 (4)
N1	0.0201 (5)	0.0227 (6)	0.0262 (6)	0.0027 (4)	0.0072 (4)	0.0031 (4)
N2	0.0190 (5)	0.0234 (6)	0.0267 (6)	0.0013 (4)	0.0062 (4)	0.0053 (4)
N3	0.0232 (5)	0.0306 (6)	0.0260 (6)	−0.0047 (4)	0.0099 (4)	−0.0026 (5)
C1	0.0241 (6)	0.0232 (6)	0.0227 (6)	0.0005 (5)	0.0093 (5)	−0.0009 (5)
C2	0.0211 (6)	0.0252 (7)	0.0233 (6)	0.0009 (5)	0.0077 (5)	0.0017 (5)
C3	0.0206 (6)	0.0268 (6)	0.0231 (6)	−0.0003 (5)	0.0063 (5)	0.0013 (5)
C4	0.0266 (6)	0.0250 (6)	0.0233 (6)	−0.0015 (5)	0.0102 (5)	−0.0005 (5)
C5	0.0296 (7)	0.0432 (8)	0.0314 (7)	−0.0132 (6)	0.0132 (6)	−0.0043 (7)
C6	0.0237 (6)	0.0348 (8)	0.0386 (8)	0.0007 (6)	0.0059 (6)	−0.0005 (6)
C7	0.0254 (6)	0.0271 (7)	0.0216 (6)	−0.0029 (5)	0.0066 (5)	0.0022 (5)
C8	0.0195 (6)	0.0289 (7)	0.0227 (6)	0.0010 (5)	0.0090 (5)	0.0033 (5)
C9	0.0247 (6)	0.0310 (7)	0.0258 (7)	0.0054 (5)	0.0089 (5)	0.0001 (5)
C10	0.0289 (7)	0.0344 (8)	0.0273 (7)	−0.0001 (6)	0.0082 (6)	−0.0029 (6)
C11	0.0212 (6)	0.0397 (8)	0.0281 (7)	0.0010 (6)	0.0053 (5)	0.0056 (6)
C12	0.0222 (6)	0.0319 (7)	0.0383 (8)	0.0060 (5)	0.0121 (6)	0.0072 (6)
C13	0.0240 (6)	0.0263 (7)	0.0326 (7)	0.0018 (5)	0.0128 (6)	0.0007 (5)

### Geometric parameters (Å, º)

**Table:**

O1—C1	1.2234 (17)	C5—H5C	0.9800
O2—C3	1.2247 (17)	C6—H6A	0.9800
O3—C7	1.2096 (17)	C6—H6B	0.9800
N1—C1	1.4094 (17)	C6—H6C	0.9800
N1—N2	1.4116 (15)	C7—H7	0.9500
N1—C8	1.4356 (16)	C8—C9	1.387 (2)
N2—C7	1.3789 (17)	C8—C13	1.3898 (18)
N2—C3	1.4236 (16)	C9—C10	1.3903 (19)
N3—C4	1.3064 (17)	C9—H9	0.9500
N3—C6	1.4607 (19)	C10—C11	1.389 (2)
N3—C5	1.4669 (18)	C10—H10	0.9500
C1—C2	1.4538 (17)	C11—C12	1.389 (2)
C2—C4	1.4019 (18)	C11—H11	0.9500
C2—C3	1.4324 (19)	C12—C13	1.390 (2)
C4—H4	0.9500	C12—H12	0.9500
C5—H5A	0.9800	C13—H13	0.9500
C5—H5B	0.9800		
			
C1—N1—N2	107.25 (10)	N3—C6—H6A	109.5
C1—N1—C8	120.96 (11)	N3—C6—H6B	109.5
N2—N1—C8	117.89 (10)	H6A—C6—H6B	109.5
C7—N2—N1	121.70 (11)	N3—C6—H6C	109.5
C7—N2—C3	122.33 (11)	H6A—C6—H6C	109.5
N1—N2—C3	110.76 (10)	H6B—C6—H6C	109.5
C4—N3—C6	126.32 (12)	O3—C7—N2	123.82 (13)
C4—N3—C5	119.34 (12)	O3—C7—H7	118.1
C6—N3—C5	114.31 (12)	N2—C7—H7	118.1
O1—C1—N1	122.81 (12)	C9—C8—C13	121.19 (12)
O1—C1—C2	129.76 (12)	C9—C8—N1	121.16 (11)
N1—C1—C2	107.42 (11)	C13—C8—N1	117.64 (12)
C4—C2—C3	134.64 (12)	C8—C9—C10	119.07 (13)
C4—C2—C1	117.00 (12)	C8—C9—H9	120.5
C3—C2—C1	108.28 (11)	C10—C9—H9	120.5
O2—C3—N2	120.52 (12)	C11—C10—C9	120.43 (14)
O2—C3—C2	133.97 (12)	C11—C10—H10	119.8
N2—C3—C2	105.51 (11)	C9—C10—H10	119.8
N3—C4—C2	132.47 (13)	C10—C11—C12	119.85 (13)
N3—C4—H4	113.8	C10—C11—H11	120.1
C2—C4—H4	113.8	C12—C11—H11	120.1
N3—C5—H5A	109.5	C11—C12—C13	120.33 (13)
N3—C5—H5B	109.5	C11—C12—H12	119.8
H5A—C5—H5B	109.5	C13—C12—H12	119.8
N3—C5—H5C	109.5	C8—C13—C12	119.12 (13)
H5A—C5—H5C	109.5	C8—C13—H13	120.4
H5B—C5—H5C	109.5	C12—C13—H13	120.4
			
C1—N1—N2—C7	161.44 (12)	C1—C2—C3—N2	−4.26 (14)
C8—N1—N2—C7	−57.85 (16)	C6—N3—C4—C2	−2.8 (2)
C1—N1—N2—C3	6.44 (14)	C5—N3—C4—C2	179.28 (14)
C8—N1—N2—C3	147.14 (11)	C3—C2—C4—N3	−9.4 (3)
N2—N1—C1—O1	170.26 (12)	C1—C2—C4—N3	174.34 (14)
C8—N1—C1—O1	31.01 (19)	N1—N2—C7—O3	10.0 (2)
N2—N1—C1—C2	−8.87 (14)	C3—N2—C7—O3	162.12 (13)
C8—N1—C1—C2	−148.12 (11)	C1—N1—C8—C9	111.30 (15)
O1—C1—C2—C4	6.4 (2)	N2—N1—C8—C9	−23.84 (17)
N1—C1—C2—C4	−174.52 (11)	C1—N1—C8—C13	−69.53 (16)
O1—C1—C2—C3	−170.80 (13)	N2—N1—C8—C13	155.33 (12)
N1—C1—C2—C3	8.25 (14)	C13—C8—C9—C10	0.6 (2)
C7—N2—C3—O2	24.0 (2)	N1—C8—C9—C10	179.72 (13)
N1—N2—C3—O2	178.78 (12)	C8—C9—C10—C11	−0.2 (2)
C7—N2—C3—C2	−156.09 (12)	C9—C10—C11—C12	−0.2 (2)
N1—N2—C3—C2	−1.27 (14)	C10—C11—C12—C13	0.0 (2)
C4—C2—C3—O2	−0.8 (3)	C9—C8—C13—C12	−0.7 (2)
C1—C2—C3—O2	175.68 (15)	N1—C8—C13—C12	−179.87 (12)
C4—C2—C3—N2	179.21 (14)	C11—C12—C13—C8	0.4 (2)

### Hydrogen-bond geometry (Å, º)

Cg1 is the centroid of the C1/C2/C3/N1/N2 ring.

**Table:**

*D*—H···*A*	*D*—H	H···*A*	*D*···*A*	*D*—H···*A*
C6—H6*A*···O2	0.98	2.08	3.016 (2)	160
C5—H5*C*···O3^i^	0.98	2.52	3.1803 (19)	124
C7—H7···O2^ii^	0.95	2.29	3.0663 (16)	139
C5—H5*B*···*Cg*1^iii^	0.98	2.98	3.8823 (18)	153

Symmetry codes: (i) −*x*+1, −*y*+1, −*z*+1; (ii) −*x*+1, −*y*, −*z*+1; (iii) −*x*+1, *y*, −*z*+1/2.
