# Supplementary figures and images for: Crystal structure of (4Z)-4-[(di­methyl­amino)­methyl­idene]-3,5-dioxo-2-phenyl­pyrazolidine-1-carbaldehyde
Source: Acta Crystallogr E Crystallogr Commun. 2015 Jun 3;71(Pt 7):o440–1. doi: 10.1107/S2056989015010038 (PMC4518909; doi:10.1107/S2056989015010038)

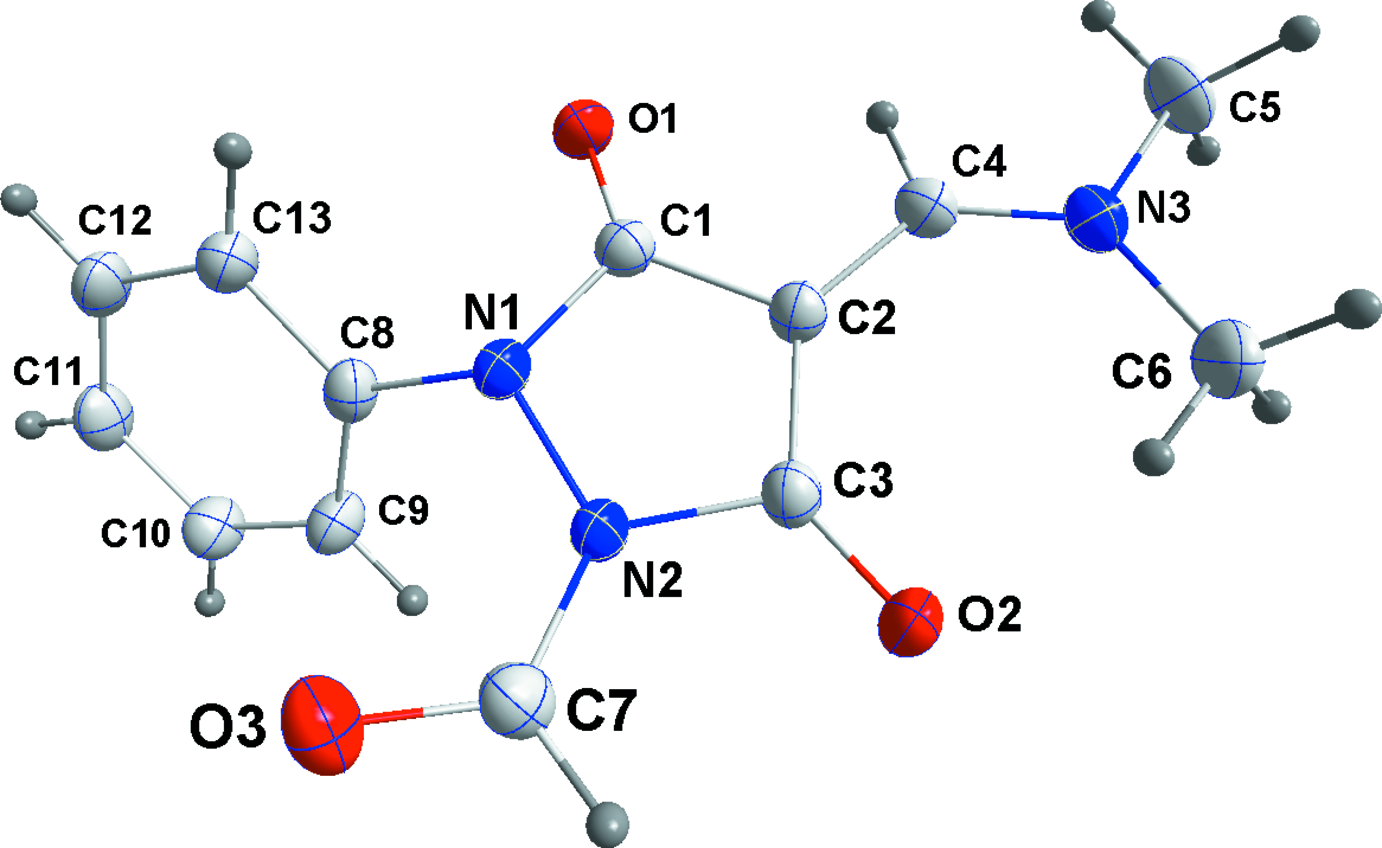

Supplement: Supplementary file 4 [file e-71-0o440-fig1.tif]

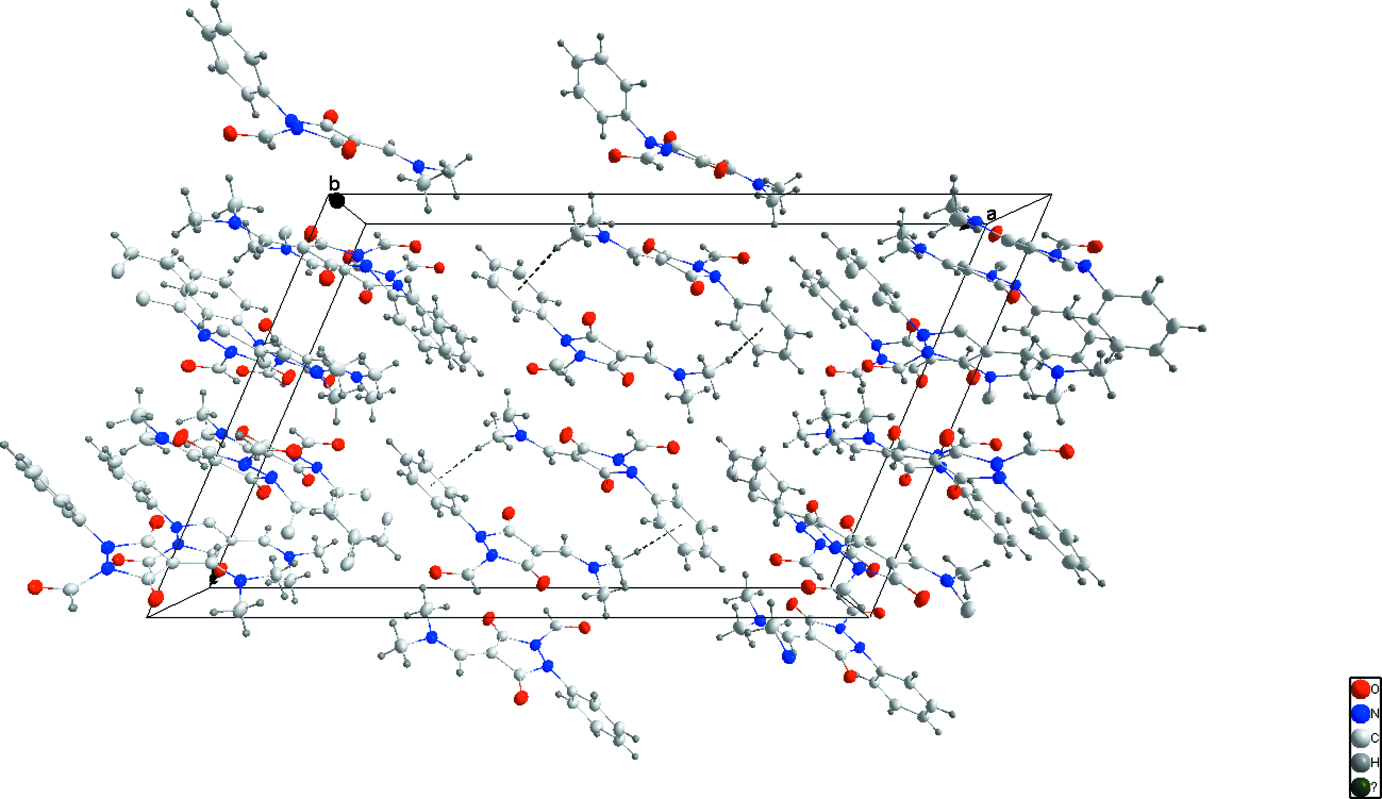

Supplement: Supplementary file 5 [file e-71-0o440-fig2.tif]
